# Poor air passenger knowledge of COVID-19 symptoms and behaviour undermines strategies aimed at preventing the import of SARS-CoV-2 into the UK

**DOI:** 10.1038/s41598-023-30654-4

**Published:** 2023-03-01

**Authors:** David. L. Jones, Jennifer M. Rhymes, Emma Green, Charlotte Rimmer, Jessica L. Kevill, Shelagh K. Malham, Andrew J. Weightman, Kata Farkas

**Affiliations:** 1 Centre for Environmental Biotechnology, School of Natural Sciences, Bangor University, Bangor, LL57 2UW Gwynedd UK; 2 SoilsWest, Centre for Sustainable Farming Systems, Food Futures Institute, Murdoch University, Murdoch, WA 6105 Australia; 3 UK Centre for Ecology and Hydrology, Environment Centre Wales, Bangor, LL57 2UW Gwynedd UK; 4 School of Ocean Sciences, Bangor University, Menai Bridge, LL59 5AB Anglesey UK; 5 Microbiomes, Microbes and Informatics Group, School of Biosciences, Cardiff University, Cardiff, CF10 3AX UK

**Keywords:** Infectious diseases, Health policy, Public health, Population screening, Diseases, Health care, Risk factors

## Abstract

Air travel mediates transboundary movement of SARS-CoV-2. To prepare for future pandemics, we sought to understand air passenger behaviour and perceived risk during the COVID-19 pandemic. This study of UK adults (*n* = 2103) quantified knowledge of COVID-19 symptoms, perceived health risk of contracting COVID-19, likelihood of returning to the UK with COVID-19 symptoms, likelihood to obey self-quarantining guidelines, how safe air travellers felt when flying during the pandemic (*n* = 305), and perceptions towards face covering effectiveness.Overall knowledge of COVID-19 symptoms was poor. Men and younger age groups (18–44) were less informed than women and older age groups (44 +). A significant proportion (21%) of the population would likely travel back to the UK whilst displaying COVID-19 symptoms with many expressing that they would not fully comply with self-isolation guidelines. Overall, males and younger age groups had a reduced perceived personal risk from contracting COVID-19, posing a higher risk of transporting SARS-CoV-2 back to the UK. Poor passenger knowledge and behaviour undermines government guidelines and policies aimed at preventing SARS-CoV-2 entry into the UK. This supports the need for stricter, clearer and more targeted guidelines with point-of-departure viral testing and stricter quarantining upon arrival.

## Introduction

The importance of air travel in facilitating the long-distance spread of COVID-19 is undisputed^1–6^. For example, it is now well established that UK citizens returning from mainland Europe (e.g. Italy, Spain and France), rather than China, were primarily responsible for initially introducing SARS-CoV-2 into the UK^7^. Based on sequencing it has been estimated that at least 1300 independently-introduced transmission lineages of the virus were introduced to the UK in early 2020, leading to the first wave of COVID-19^7^. Based on this, we estimate that infected passengers entering the UK represented ca. 0.02% of the 8 million passengers arriving during this period. This was, however, notably lower than the estimated 1.3% of infected international passengers that left Wuhan at the start of the pandemic and which fuelled the global spread of the disease^8^. At the beginning of the second COVID-19 wave in Europe, in-flight transmission of SARS-CoV-2 between passengers was also documented in a flight from Greece to Ireland, where the attack rate was 10–17%^9^. This transmission occurred despite the use of face coverings and the implementation of social distancing measures. A range of modelling and epidemiological case studies has also confirmed that one infected person on a flight could transmit the disease to other passengers throughout the plane^10^. This is not helped by the close proximity of other passengers, intrinsic air circulation patterns and closely confined and high frequency use toilet facilities. The potential for importing and transmitting SARS-CoV-2 on aircraft mirrors the findings for other respiratory viral diseases (e.g. influenza;^11,12^). In addition, the potential for transmission within crowded airport terminals has also been demonstrated for other respiratory pathogens (e.g. adenovirus;^13^) suggesting that a single individual carrying the disease may infect multiple people all travelling to different destinations.

Although closure of international flights has inevitably helped contain the spread of the disease, it has come at a substantial cost^14–16^. For example, COVID-19 restrictions on air travel has led to major economic and job losses in many countries^17,18^. There is therefore an urgent drive from the travel industry to re-open international air routes post-pandemic. However, this must be done in a socially responsible, practical and economic way that will ensure protection of public health. Key to this is effective disease control and surveillance. This clearly requires the development of practical, cost-effective and socially acceptable surveillance technologies but also necessitates a good knowledge of individual attitudes to COVID-19 and their behaviour before, during and after air travel. From one survey of the public acceptance of quarantining measures it was concluded that public support is vital for any program involving quarantine and isolation^19^. Further addressing challenges and barrier to enlist the support of the public is essential to optimize compliance^20,21^.

Appraisal of airport entry screening measures have shown that it is highly resource demanding^22^ and often ineffectual^4,23^. Although a range of strategies are now in place for national disease surveillance (e.g. contact tracing, self-reporting apps, targeted and untargeted swab, testing, seroprevalence), we still lack ways to reliably estimate rates of disease entry from overseas travellers. Based on the known trans-national importation of new variants of SARS-CoV-2 into the UK (e.g. beta, gamma, theta, omicron), it is clear that current surveillance strategies remain inadequate both at the point of departure and the point of entry. This is either because (i) current technologies lack scientific credibility (e.g. thermal imaging gates), (ii) are not cost-effective for mass deployment, (iii) are subject to error (e.g. lateral flow devices, swab testing), (iv) fail to capture recently acquired infections (e.g. those acquired within hours of departure), (v) are not available at the point of departure, (vi) cannot capture infections acquired during travel (e.g. in transit lounges or on the flight), or (vii) solely rely on self-reporting which fails to capture asymptomatic, pre-symptomatic, mildly symptomatic individuals and those knowingly concealing symptoms^24,25^. This is supported by an ECDC study which estimated that ca. 75% of infected individuals from China arrived at their destination undetected^26^. To help mitigate this, many countries have implemented policies of quarantining passengers for 10–14 days upon arrival.

Clearly, self-quarantining relies on individual compliance if it is to be effective. In particular this includes obedience and/or agreement to follow quarantining polices. It has been suggested that obedience involves respect of implicit and explicit rules and that is a socially learned behaviour^27^. In contrast, disobedience is suggested to be a behaviour where an individual takes a conscious stance against formalized laws or implied social norms^27^. Therefore, if individuals refuse to comply with quarantining policies, due to obedience being a learned behaviour from childhood, then these individuals may also be less likely to follow other health advice put forward to mitigate the spread of COVID-19 (e.g. mask wearing), posing further risk to themselves and others.

With a focus on UK air travellers and those that have flown during the pandemic, the primary aims of this study were to evaluate how human behaviour associated with travel can increase the risk of spreading COVID-19. We aimed to (i) evaluate passenger knowledge of COVID-19 symptoms, (ii) their attitudes to catching COVID-19, (iii) evaluate their likelihood of returning back to the UK if they, or a member of their family, were ill, (iv) evaluate their perceived safety during recent air travel, and (v) the likelihood that they would self-quarantine for the full period on return to the UK.

## Materials and methods

### Study design

We commissioned the ESOMAR accredited market research company YouGov (YouGov Ltd., London, UK;^28^) to carry out this cross-sectional survey, between the 22nd to 23rd October, 2020. Following a full national lockdown introduced in March 2020, at the time of this study the UK remained under tight COVID-19 restrictions that limited social mixing^29^ although, specific guidance varied between England, Wales, Scotland and Northern Ireland. Participants (*n* = 2103) were recruited from YouGov's online research panel (*n* = 800,000 + UK adults) and were eligible if they were aged 18 years or older and living in the UK and had undertaken foreign air travel. Comparisons of opt‐in internet panels with traditional stratified random sample interview and random digit dial techniques conclude that the biases introduced by this methodology are small, and in general are more than offset by the much larger sample sizes the internet‐based methodology permits^30^. The random error on a sample of 2,000 individuals is estimated to be up to 2%. Quota sampling was used, based on age, gender and Government Office Region, to ensure that the sample was broadly representative of the UK general population. All participants provided sociodemographic variables and none were excluded from the subsequent analysis. Participants were invited to participate in the survey by an email with the subsequent survey conducted on-line via the YouGov data portal. Active sampling restrictions were put in place to ensure that only people contacted and registered with YouGov were allowed to participate. Participants were provided with a summary of the surveys purpose and were informed that data collection, storage and analysis would follow the Data Protection Act 2018. Individuals were then asked to consent prior to survey participation.

### Questionnaire

The survey consisted of 15 closed-ended questions, with 7 of the questions addressing issues associated with travelling by air and 8 questions addressing specific demographic topics. The questionnaire was designed by the research team, consisting of environmental microbiologists, public health specialists and social scientists, based on the study objectives and incorporating information from previous studies on same topic. The draft questionnaire was then tested on an expert panel, a panel of non-experts, a local ethics committee and finally refined by YouGov prior to deployment. First, perceived risks, concerns, and subjective knowledge of COVID-19 symptoms were measured using 16 options that included 14 actual symptoms and 2 which were not. Other questions about perception and risk were measured by statements with a 5-point Likert scale (e.g. strongly disagree to strongly agree). The survey questions and responses are available as an open-access data archive on the Zenodo repository, https://doi.org/10.5281/zenodo.6958979.

### Personal characteristics

We asked participants to report their age, gender, social grade, employment status, highest educational or professional qualification and marital status. We also asked whether there was a child in their household, what social media/messaging platforms they had used in the last month (Facebook, Twitter, LinkedIn, Instagram, Snapchat WhatsApp, Skype), whether they had travelled abroad by plane since the start of the COVID-19 pandemic and whether they had ever tested positive for COVID-19. Participants were asked for their postcode to determine indices of multiple deprivation (IMD) and their social grade. The number of respondents for the key demographic variables are summarised in Table 1.

**Table 1:** Respondent key demographic variables. Note -respondents were able to respond with more than one choice of social media platform used.

	Number	% of total
Age		
18–24	210	9.9
25–34	311	14.8
35–44	403	19.2
45–54	365	17.4
55 +	814	38.7
Gender		
Male	1008	47.9
Female	1095	52.1
Social grade		
A, B, C1	1230	58.5
C2, D, E	873	41.5
Marital status		
Married	931	44.3
Living as married	257	12.2
Separated/divorced	198	9.4
Widowed	89	4.2
Never married	616	29.3
Parent/guardian		
Yes	1207	57.4
No	896	42.6
Social media		
Facebook	1478	22.1
Twitter	756	11.3
LinkedIn	373	5.6
Instagram	774	11.6
Snapchat	317	4.8
Facebook Messenger	1337	20.0
WhatsApp	1426	21.4
Skype	215	3.2

### Ethics

This study was performed in accordance with relevant guidelines and regulations, where ethical approval for this study was granted by the Bangor University College of Environmental Sciences and Engineering Ethics Committee (Approval Number: COESE2020EG01A).

### Analysis

Any percentages calculated on bases fewer than 50 respondents (< 2.3% of the total) were included with the caveat that they may not represent a true cross-section of the target population and should be used as indicative only. Comparisons between groups was made using chi-squared tests using *P* < 0.05 as the cut-off for statistical significance.

## Results

### Recognition of COVID-19 symptoms

**Figure 1:**
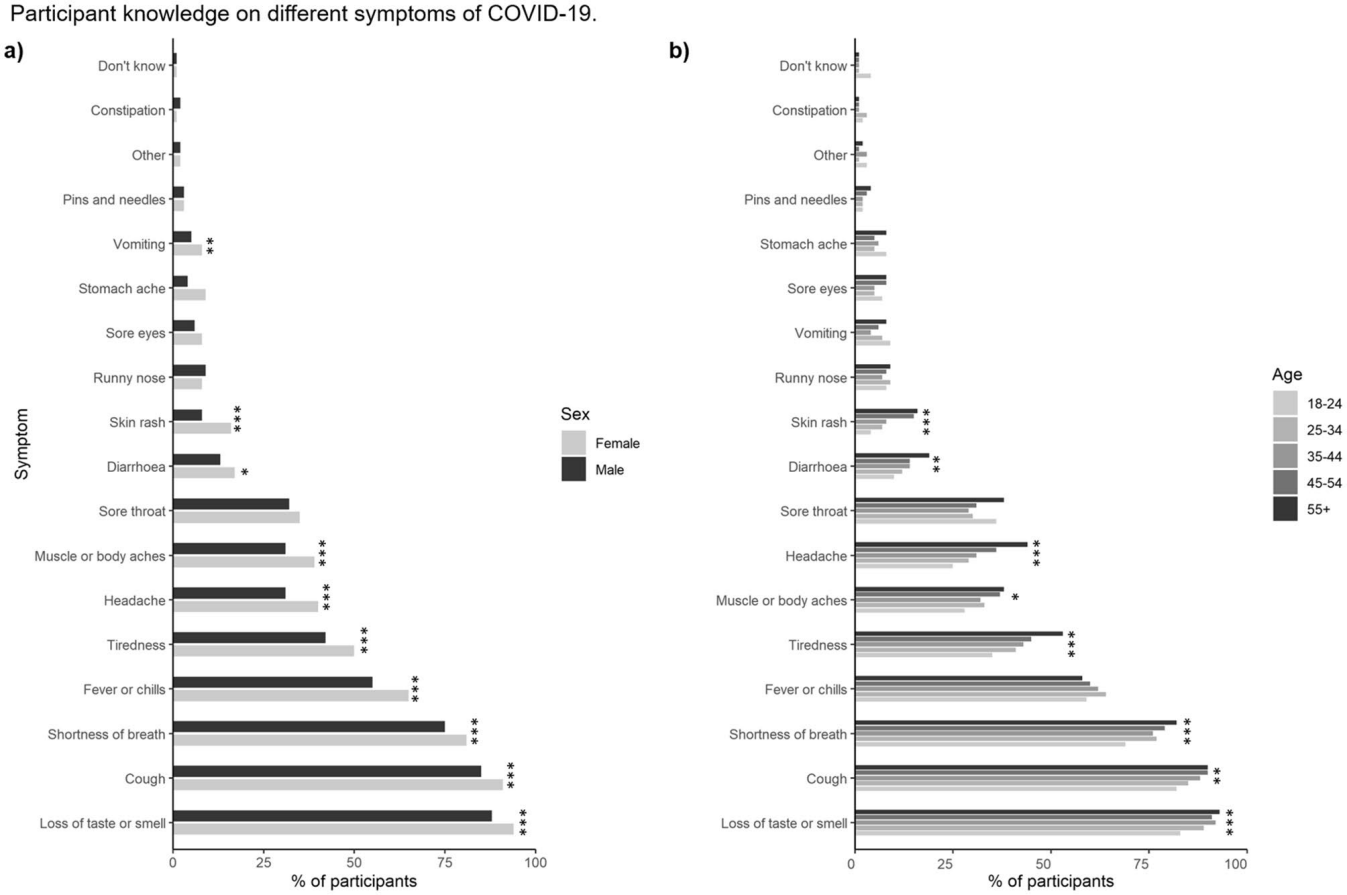
Proportion of participants stratified by either (**a**) gender, or (**b**) age (*n* = 2103). *, **, and *** represent significant differences between the gender or age categories for a particular symptom at the *P* < 0.05, *P* < 0.01 or *P* < 0.001 level, respectively.

Overall, there was good knowledge of the main symptoms of COVID-19 (e.g. fever, cough, shortness of breath) among the respondents (Fig. 1a). In contrast, other symptoms associated with the onset of COVID-19 were not recognized by the majority of respondents (e.g. skin rash, muscle and body aches, diarrhoea, headache, nausea and vomiting). The two symptoms not typically associated with COVID-19 (nerve pain and constipation) were only highlighted by ca. 2% of respondents. For the 10 symptoms where the response rate was greater than 10% (i.e. *n* > 250), female respondents were better able to recognize the actual COVID-19 symptoms by 25 ± 9% in comparison to the male cohort (*P* < 0.001). In addition, for the same top 10 ranked symptoms, 9 showed an increased recognition of symptoms with age (Fig. 1b; *P* < 0.05). When comparing the cohort with least risk to developing severe COVID-19 symptoms (ages 18–24) to those in the older, more susceptible cohort (age 55 +), the older generation were on average 66 ± 29% better at recognizing the symptoms (*P* < 0.001). No significant effect of social class (UK Office for National Statistics classes A, B, C1 versus C2, D, E; see Supplementary Information Table S1 for details) on COVID-19 symptom recognition was observed (*P* = 0.65). The responses of adults with and without children was also very similar, although those with children (< 18 years in age) were on average 9 ± 3% better at recognizing the symptoms. The variation in COVID-19 symptom recognition was not associated with media platforms used by the respondents in the last month (all *P* > 0.05).

**Figure 2:**
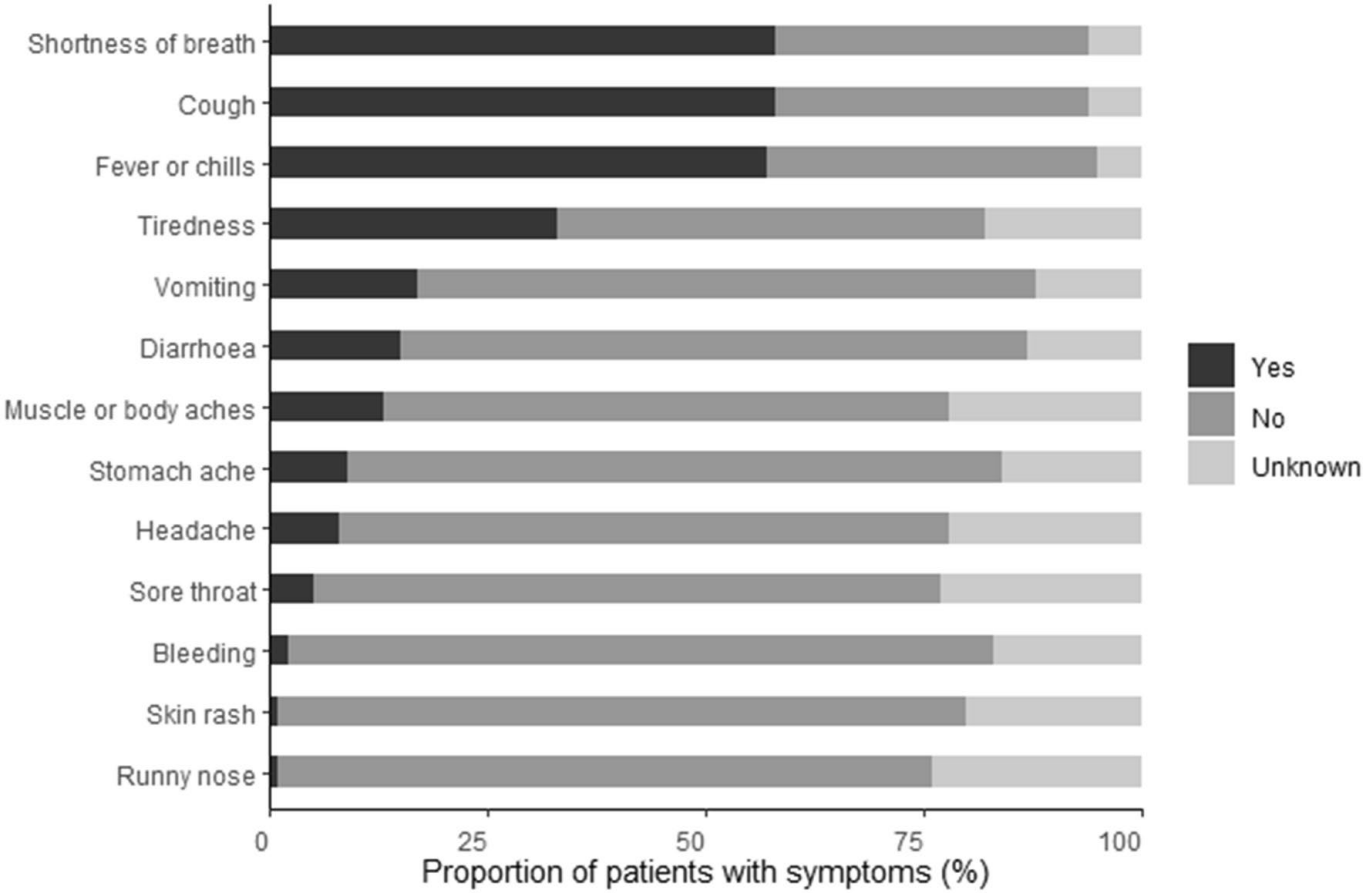
Actual symptoms of COVID-19 experienced by infected individuals in the UK at the time the survey was conducted. (*Source*: Welsh Government—Technical Advisory Group). Unknown refers to instances where it was not possible to state whether the symptom was experienced or not.

The actual reported symptoms of COVID-19 by individuals in the UK at the time of the survey^31^ is shown in Fig. 2. Overall, there is a partial agreement about the most common perceived (Fig. 1) and actual (Fig. 2) COVID-19 symptoms, however, there are notable exceptions including vomiting, abdominal pain, diarrhoea and fatigue which were not widely recognized as symptoms by the majority of individuals, particularly men and the younger age groups.

### Perception of the risk of catching COVID-19

**Figure 3:**
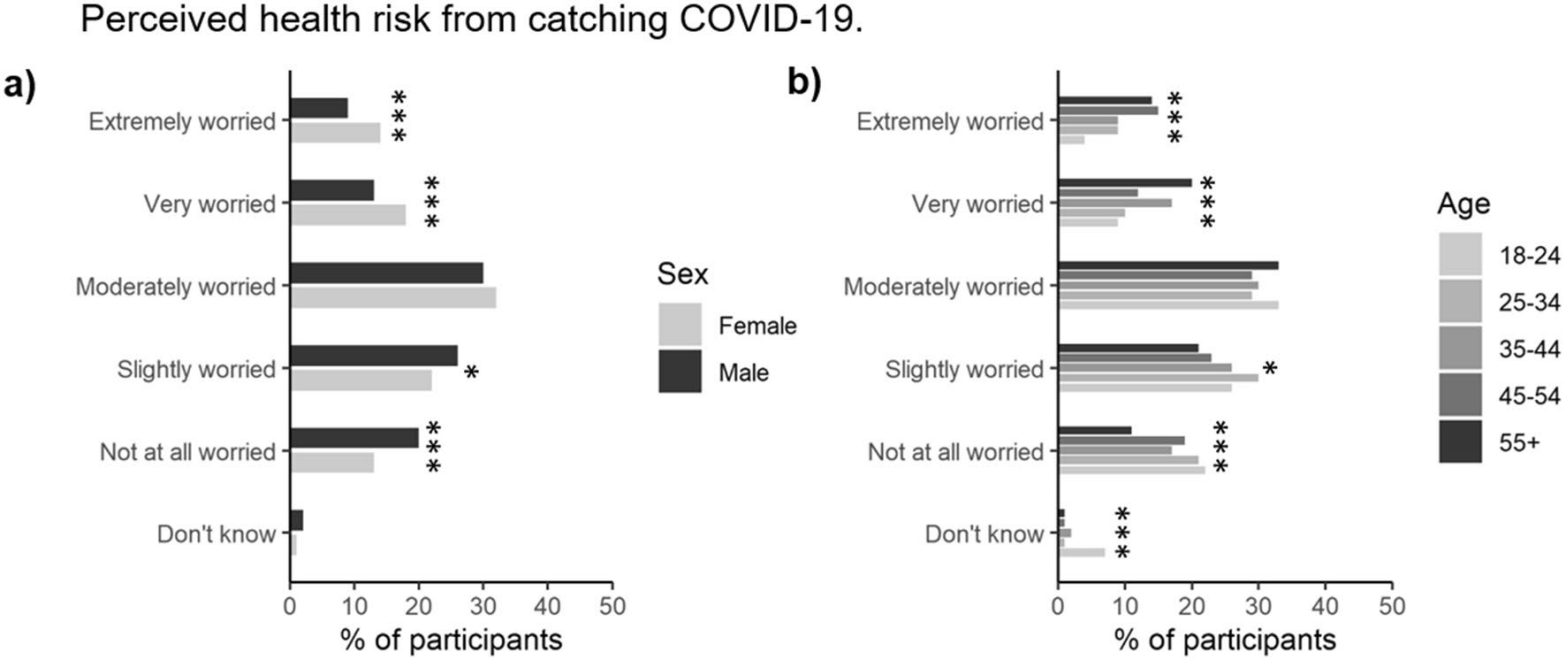
Proportion of participants stratified by either (**a**) gender, or (**b**) age (*n* = 2103). *, **, and *** represent significant differences between the gender or age categories for a particular symptom at the *P* < 0.05, *P* < 0.01 or *P* < 0.001 level, respectively.

Of those who answered (*n* = 2103), 17% of respondents were not worried about catching COVID-19, 56% expressed some concern and 27% exhibited strong concern about catching the disease (Fig. 3). This was not greatly affected by social grade, however, greater concern was expressed in females (Fig. 3a, *P* < 0.001) and older people relative to those in the youngest age group (34 vs. 13%) and also by parents with children relative to those without (31 vs. 22%). Of those surveyed, 1.3% (*n* = 27) had previously tested positive for COVID-19, while a further 10.3% believed they had contracted COVID-19 but had never been formally tested, with the remainder not knowingly having contracted the disease. The number of confirmed or suspected positive cases was not associated with differences in gender, age, social class or the presence of children in the household.

### Perceived likelihood of flying back to the UK while showing signs of illness and the likelihood of quarantining (self-isolation) upon landing in the UK

**Figure 4:**
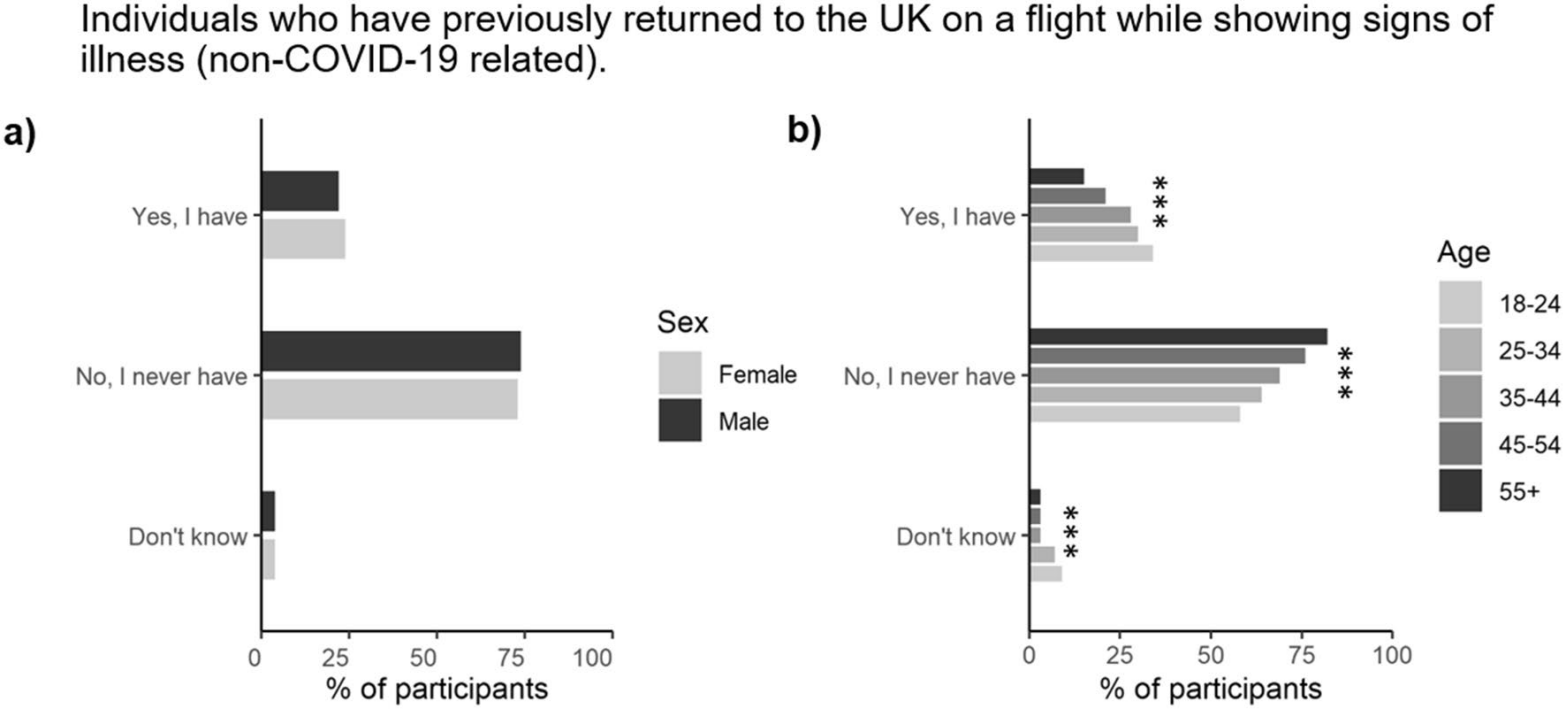
Proportion of participants stratified by either (**a**) gender, or (**b**) age (*n* = 2103). *, **, and *** represent significant differences between the gender or age categories for a particular symptom at the *P* < 0.05, *P* < 0.01 or *P* < 0.001 level, respectively.

When asked about their previous experience of returning back to the UK on an international flight, 23% of respondents indicated that they had previously boarded a flight while feeling ill (e.g. feeling sick, diarrhoea, headache etc.; Fig. 4). Although not affected by social grade or gender, a greater proportion of the younger age groups (ages 18–44, *n* = 833) had travelled while ill in comparison to those in the older age groups (ages 44 + , *n* = 1076, *P* < 0.001). Travelling while ill was also more frequent in households without children (29 vs 18%).

**Figure 5:**
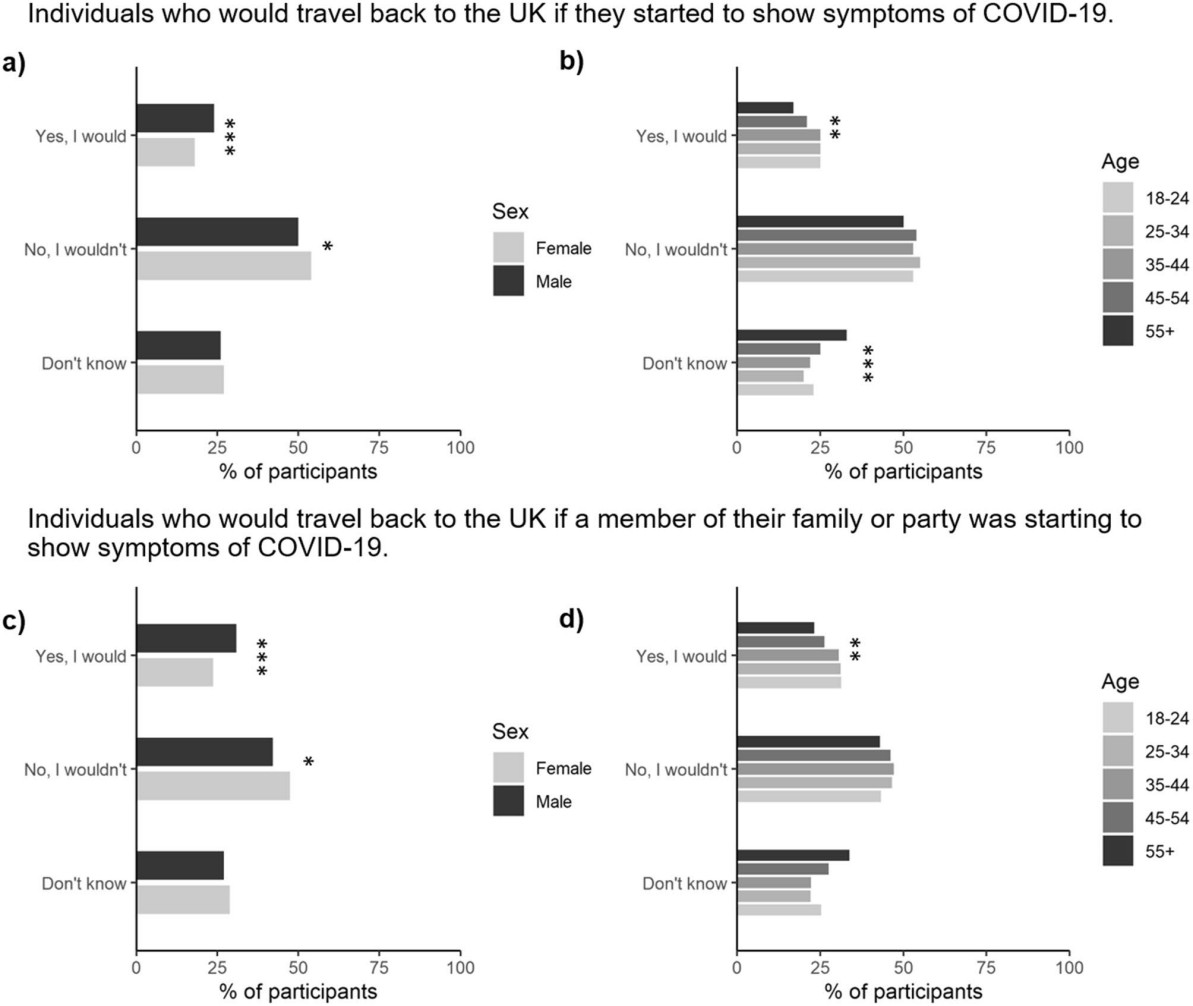
Proportion of participants stratified by either (**a**) gender, or (**b**) age (*n* = 2103). *, **, and *** represent significant differences between the gender or age categories for a particular symptom at the *P* < 0.05, *P* < 0.01 or *P* < 0.001 level, respectively.

In the hypothetical situation that an individual started to express potential symptoms of COVID-19, we asked their likelihood of returning on their scheduled flight. Overall, 21% said they would, 52% said they would not and 27% of individuals indicated that they were unsure (Fig. 5a,b). Overall, slightly more men said they would potentially travel back with COVID-19 symptoms relative to women (24 vs. 18%, *P* < 0.001). A return to the UK while expressing COVID-19 symptoms was also higher in the younger age groups (ages 18–44) relative to those in the older age groups (ages 44 +, *P* < 0.001) while social grade and the presence of children in households proved not to be significant.

When individuals were asked whether they would fly home with another person who might by exhibiting potential COVID-19 symptoms (even though they themselves were not), the responses were generally similar to responses if they had symptoms, with 27% saying they would still travel home, while 45% would not and 28% of individuals remained unsure (Fig. 5c,d).

**Figure 6:**
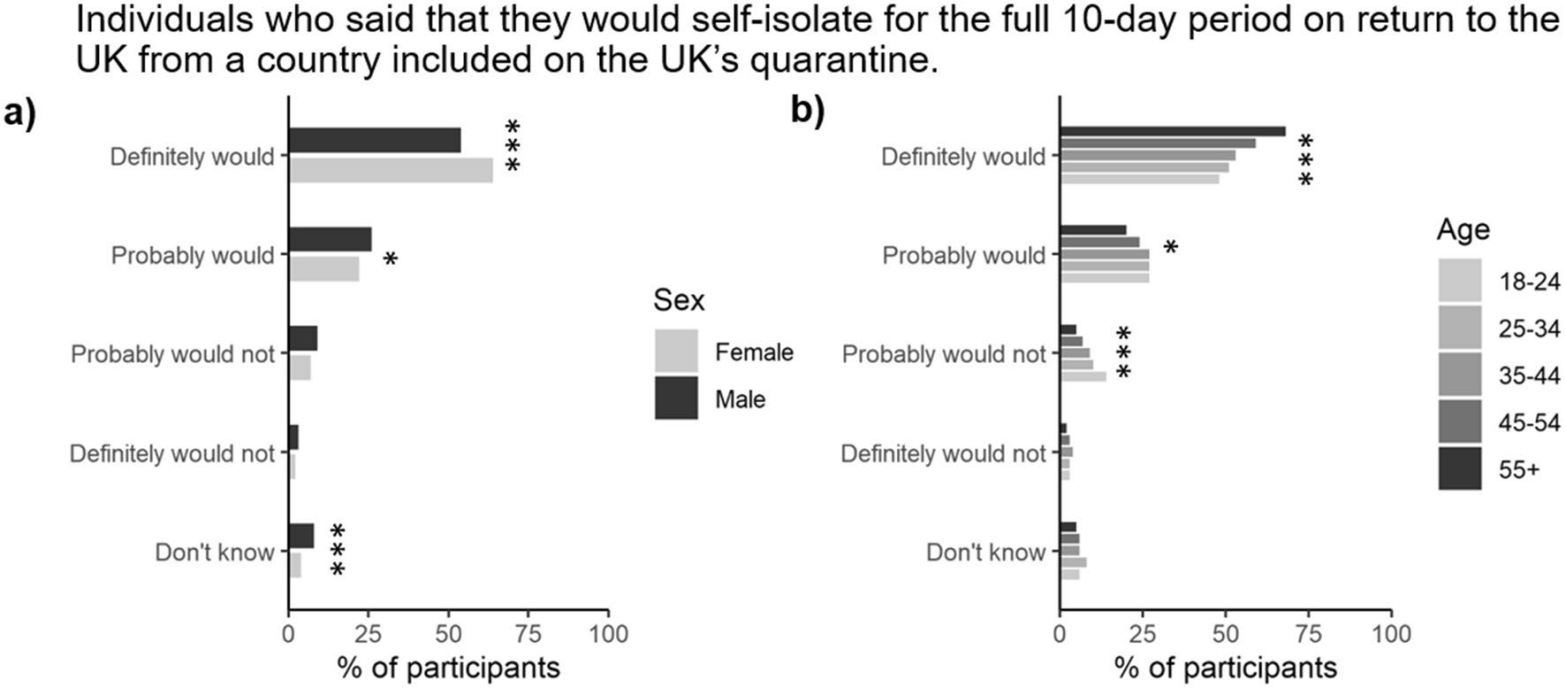
Proportion of participants stratified by either (**a**) gender, or (**b**) age (*n* = 2103). *, **, and *** represent significant differences between the gender or age categories for a particular symptom at the *P* < 0.05, *P* < 0.01 or *P* < 0.001 level, respectively.

To better understand the likelihood that an individual would self-quarantine following return from a country on the UK government’s quarantine list, 83% of individuals reported that they would probably quarantine for the full 10-day period, while 10.2% said they would not (Fig. 6). These responses were stratified by age and gender (Fig. 6a), but not social grade or the presence of children in the household. Females were more likely to obey government guidance than men (*P* < 0.001). The younger generation were also more likely to break the self-isolate guidance (17%) relative to the older population (6%).

### Personal safety while flying during the COVID-19 pandemic

**Figure 7:**
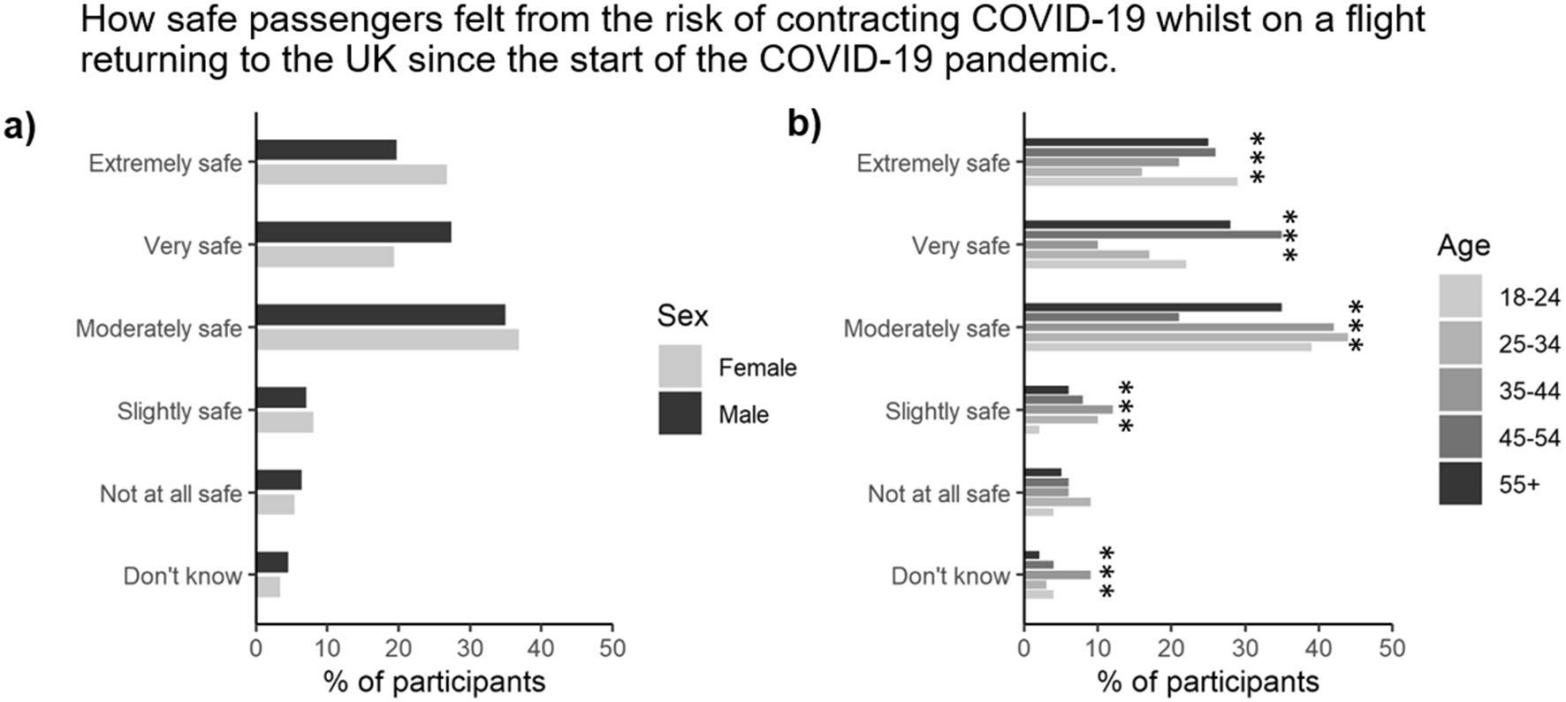
Proportion of participants stratified by either (**a**) gender, or (**b**) age (*n* = 305). *, **, and *** represent significant differences between the gender or age categories for a particular symptom at the *P* < 0.05, *P* < 0.01 or *P* < 0.001 level, respectively.

Of the total number of respondents polled, 15.2% (*n* = 305) of them indicated they had flown since the start of the COVID-19 pandemic. The greatest numbers of flights were taken by the youngest age group (age 18–24) and the more affluent social grades (A, B, C1). Of these, 47% expressed that they felt safe from potentially catching COVID-19 during the flight while 13% indicated that they did not (Fig. 7). These levels were only affected by any of the demographic categories analysed here.

**Figure 8:**
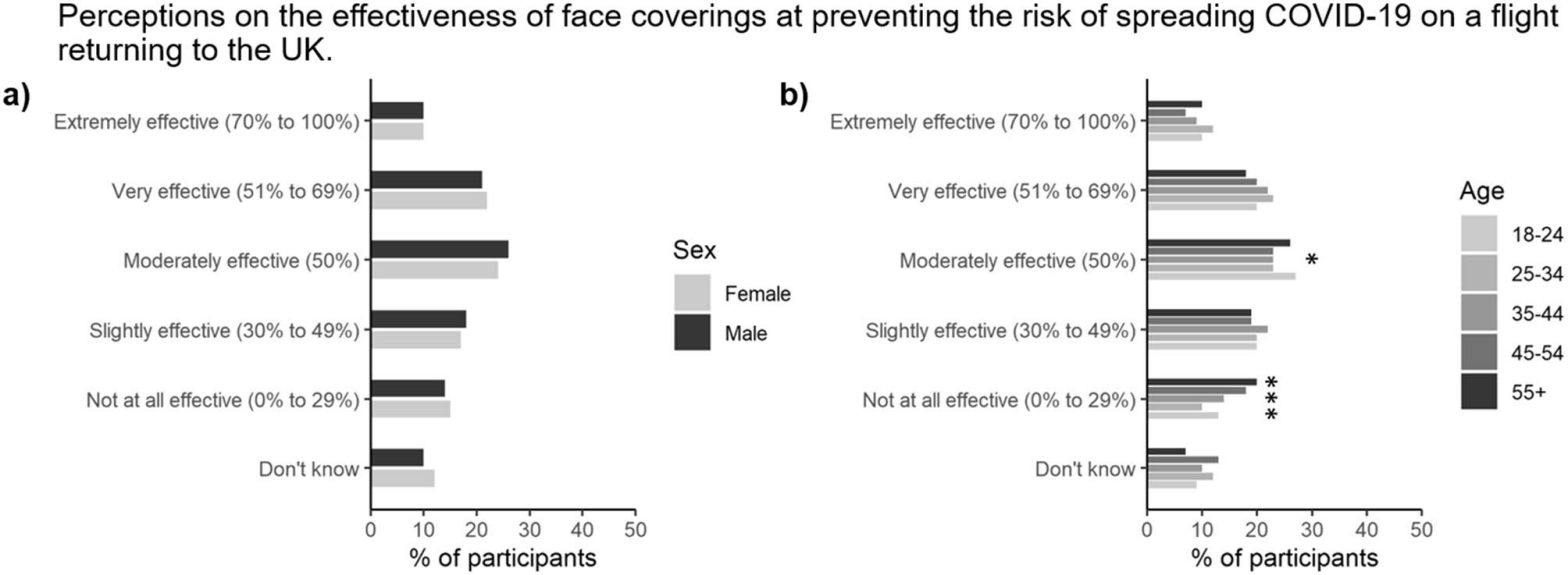
Proportion of participants stratified by either (**a**) gender, or (**b**) age (*n* = 2103). *, **, and *** represent significant differences between the gender or age categories for a particular symptom at the *P* < 0.05, *P* < 0.01 or *P* < 0.001 level, respectively.

When asked about the effectiveness of containing the spread of COVID-19 on the flight with face coverings, the respondents were equally split with 32% reporting that they were effective, 36% only partially effective, and 32% reporting that they were ineffective (Fig. 8). These views were not strongly influenced by any of the demographic categories analysed here. Further analysis revealed that 93% of individuals would wear face masks on a plane, but of these 31% would only do it if it was mandatory (data not presented). These proportions were not influenced by social grade or gender, however, the over 55 age group were more likely to wear a face mask whether it was mandatory or not (72%) relative to the other age groups (57 ± 2%).

## Discussion

### Air passenger knowledge of COVID-19 symptoms

Overall, we found that an individual’s knowledge of the range of symptoms associated with COVID-19 was mixed. Respondents had a good knowledge of the main symptoms of COVID-19 (e.g., fever, cough, shortness of breath) but did not recognise other symptoms (e.g., skin rash, muscle and body aches, diarrhoea, headache, nausea and vomiting). This lack of awareness increases the risk of spreading COVID-19 as individuals are unable to effectively self-diagnose a potential COVID-19 infection and act appropriately to mitigate the spread of COVID-19 when travelling. This poses a particular risk early on within an infection where some point-of-departure detection technologies (e.g. lateral flow devices) are not reliable^32,33^. It is also important as a high proportion of individuals who contract COVID-19 can experience mild or less recognised symptoms (Fig. 2;^34^) and in some cases in isolation of the more common symptoms^35^. It is therefore vital to improve the public’s knowledge on symptoms through media reports and education as this has been shown to positively alter behaviour during a disease outbreak^36^. In this instance, it will allow people to assess their symptoms effectively and subsequently foster more responsible travel behaviour during a pandemic. However, this is particularly challenging as distinguishing COVID-19 symptoms from other common food poisoning agents (e.g. Campylobacter, Salmonella), viral illnesses (e.g., rhinovirus, norovirus, enterovirus), pharmaceutical use and excessive alcohol consumption can be difficult^37^. There is evidence to show that free testing facilities can effectively alter behaviours to reduce community transmission^38^; we therefore highly recommend that people are given easy access to testing facilities to allow individuals to distinguish between COVID-19 and other common illnesses when there is uncertainty around the symptoms being experienced.

### Likely compliance with government strategies to minimize disease spread

A major risk for COVID-19 entry into the UK is a lack of compliance with government mitigation strategies aimed at reducing the spread of COVID-19. Here, we found that 21% of respondents would not follow travel advice where the public are asked to not travel when expressing COVID-19 symptoms. Furthermore, 10% of respondents would not comply with the government self-isolation guidelines upon entry into the UK. Understanding these behaviours is integral in providing ways in which health planners and policy makers can encourage compliance. Although this study does not explore these factors, it is likely that these behaviours are driven by similar factors identified by studies that focus on general compliance behaviours during a pandemic, which are not specific to travel. These include a lack of perceived risk to the virus^39^, financial worries (e.g., not understanding the financial support available to them, the need to work, worrying about supporting their families), social and cultural pressures and mental wellbeing etc.^40,41^.

### In-flight COVID-19 transmission risks associated with passenger perceptions and behaviours

Multiple factors can contribute to the cumulative risk of contracting COVID-19 from air travel^42^. Mitigation strategies have been implemented internationally to reduce the risk of spreading COVID-19 by air travel (e.g., mandatory face coverings, installation of HEPA air filters), however, several risks that are more closely aligned to one’s perception and/or behaviour to flying during a pandemic has the potential to further increase risk despite implemented mitigation strategies. Evaluating the risk perception of air passengers that travelled during the COVID-19 pandemic highlighted that a high proportion of individuals felt safe from contracting COVID-19 during air travel. This suggests that non-pharmaceutical interventions to reduce disease transmission were being correctly practiced during their flights.

According to Anderson et al.^43^, handwashing with soap is the most effective way to mitigate the spread of infectious diseases. Despite this, globally, the benefits of hand hygiene are poorly practiced^44^. This is particularly problematic in cramped aircraft bathrooms where there is a high risk of cross contamination due to the large number of touch surfaces^45^. Additionally, Suen et al.^46^ found that many individuals overlook the actual importance of washing their hands (hand hygiene) when taking part in activities where they would be required to wash their hands. In support of this, our research found that only 84% of participants would wash their hands following the recommended guidelines for COVID-19. Furthermore, they proposed that female respondents generally have better knowledge regarding hand hygiene which supports the findings from this study where it was mainly the women and the older participants (44 +) that stated they would follow COVID-19 recommended guidelines with regards to washing hands. It is recommended that when promoting hygiene as a guideline, that more gender specific material be used to ensure that the importance of hygiene, whether it be hand hygiene or hygiene in general is understood and adopted by all individuals to sustain improvement in hygiene practices^47^.

### Gender differences in COVID-19 awareness and risk perception

In agreement with Kamenidou et al.^48^, we found that women were generally better at identifying COVID-19 symptoms compared to the male participants. We believe that previously reported differences between males and females such as, men having a lower risk perception^49^, a disbelief that COVID-19 is contagious^50^ and a sense of COVID-19 immunity^51^ are likely to also contribute towards the observed differences between age groups and adults with or without children.

Our research supports previous findings by Bass et al.^19^ that women would be more willing to adhere to various levels of quarantine compared to men. Furthermore, our research agrees with Galasso et al.^52^ that women were more likely to perceive COVID-19 as a very serious health problem making them more likely to agree and comply with government guidelines. Additionally, Bass et al.^19^ found that respondents who were older than 65 were more willing than younger respondents to stay at home when government guidelines were in place to do so which also agrees with our findings. Overall, our findings suggest that males and younger ages groups have a reduced perceived personal risk from contracting COVID-19 and our results suggest that they pose a higher risk of transporting SARS-CoV-2 back to the UK and around the UK due to lack of willingness to co-operate with isolation guidelines.

### Age differences in COVID-19 awareness and risk perception

We found a higher proportion of older generations (aged 44 +) and adults with children were able to recognise COVID-19 symptoms compared to younger age groups (18–44) and adults without children, respectively. Our findings suggest that males, young adults, and adults without children pose a higher risk in spreading COVID-19 infections and that information needs to be created to target each segmentation in a more unique way. Similar findings have also been reported for the non-air travellers in Africa^53^.

Our findings agreed with previous research that younger participants (under the age of 25) were unable to identify many symptoms connected to COVID-19 and that they would be less likely to pay the required attention when symptoms appeared^48^. The aforementioned study suggests that the younger population are more interested in solving the issues that COVID-19 presents rather than focusing on how they can prevent the spread. This is compounded by negative media representation of the role of younger generations in spreading COVID-19 and their over-reliance on social media for information^54^. Again, this suggests that information needs to be targeted to segmented age groups to ensure the correct information is retained and processed.

With regards to following guidelines set by the government, our findings suggest that the younger generation were more likely to break the self-isolation guidance (17%) relative to the older population (6%). In contrast, research conducted in Italy by Ceccato et al.^55^ found no age-related differences in compliance with the regulation. They did, however, identify that the 20% who did not follow the strict guidelines, were the same participants that had less confidence in the public health information being provided. These findings strengthen the importance of not only the delivery of information, but also the reliability of the information being presented.

### Conclusions

Based on this national survey of air passenger behavioural patterns and individual perceptions of COVID-19, alongside known importation of new SARS-CoV-2 variants into the UK, we conclude that past and current government guidelines and policies are insufficient to prevent the frequent entry of SARS-CoV-2 into the UK. This is supported by information provided by the English *Test and Trace* and Welsh *Test*, *Trace*, *Protect* programmes which still show high rates of SARS-CoV-2 carriage among incoming passengers (1–7.5% of total;^56^) and also non-compliance with self-quarantining rules which have subsequently led to major outbreaks (Public Health Wales, pers. comm.). Our recommendation supports the imposition of stricter guidelines to ensure complete compliance with point-of-departure PCR-based COVID-19 testing and stricter quarantining on arrival for UK citizens returning from overseas^57^ A limitation of this study was that it was only confined to UK citizens. It would therefore be desirable to repeat the study to evaluate the perceptions of non-UK citizens where additional socioeconomic and cultural barriers may prevent non-compliance and enhance the risk of disease entry into the UK. Typically, pre-pandemic these account for ca. 59% of all air travellers entering the UK^58^, although it should be noted that these have been subject to compulsory quarantining in government facilities, rather than self-quarantining at home like UK citizens. Despite the well understood risks associated with air travel and disease transmission (e.g. confined space and poor airflow) and that mitigations have been implemented to reduce these risks (e.g. compulsory face coverings), we highlight additional risks that should be considered that are associated with an individual’s perception and related behaviours towards COVID-19 that can jeopardise existing strategies to reduce the spread of SARS-CoV-2.

In terms of future pandemics, our work suggests that despite repeated messaging from national agencies, greater emphasis is needed to ensure that all individuals understand the importance of following public health guidelines and how self-imposed quarantining and extended hygiene can help to reduce infection spread. Additionally, the presentation of disease symptoms (both main symptoms and onset) needs to be addressed in ways in which they are understood and adopted by all individuals to reduce the chance of spreading infection. Different ways of communication are also needed particularly to reach younger generations travelling by air.

## Supplementary Information

Supplementary Information.

## Data Availability

The datasets generated and/or analysed during the current study are available in the Zenodo repository, https://doi.org/10.5281/zenodo.6958979.
