# Supplementary material for: Poor air passenger knowledge of COVID-19 symptoms and behaviour undermines strategies aimed at preventing the import of SARS-CoV-2 into the UK
Source: Sci Rep. 2023 Mar 1;13:3494. doi: 10.1038/s41598-023-30654-4 (PMC9976683; doi:10.1038/s41598-023-30654-4)
Supplement: Supplementary file 1 — Supplementary Information. [file 41598_2023_30654_MOESM1_ESM.docx]

**Supplementary Information**

Approximated Social Grade with its six categories A, B, C1, C2, D and E is a socio-economic classification produced by the ONS (UK Office for National Statistics) by applying an algorithm developed by members of the MRS Census & Geodemographics Group. It applies to every Household Reference Persons (HRP) aged 16 to 64. Social Grade based on the census 2011 data is available for all four UK countries (England, Wales, Northern Ireland and Scotland).

**Table S1.** Social grade categories used in the questionnaire.

| Social grade | Description | % of UK population |
| --- | --- | --- |
| AB | Higher & intermediate managerial, administrative, professional occupations | 22.17 |
| C1 | Supervisory, clerical & junior managerial, administrative, professional occupations | 30.84 |
| C2 | Skilled manual occupations | 20.94 |
| DE | Semi-skilled & unskilled manual occupations, Unemployed and lowest grade occupations | 26.05 |
